# The post-pandemic transformation in Pathophysiology teaching strategies

**DOI:** 10.3389/fmed.2026.1738205

**Published:** 2026-04-22

**Authors:** Huiling Lu, Xin Li, Lingli Zhang, Min Xin, Qiuhui Wu, Zhen Wu, Huimin Peng, Jian Chen, Ningxia Zhu

**Affiliations:** 1 Department of Pathophysiology, Guilin Medical University, Guilin, Guangxi, China; 2 Department of Physiology, Guilin Medical University, Guilin, Guangxi, China

**Keywords:** blended learning, case-based learning, integration, Pathophysiology, post-pandemic

## Abstract

The pandemic has fundamentally altered the way that medical students acquire knowledge, making blended learning the predominant student-centered teaching model. This approach has demonstrated positive effects on developing key competencies in clinical medical students. Furthermore, research indicates that case-based learning (CBL) and integrated teaching are beneficial attempts to achieve the above educational goals. In this study, students from the grades of 2020 and 2021 were provided with Massive Open Online Course (MOOC) resources. A blended learning approach combining CBL with cross-chapter integration in Pathophysiology was implemented to construct an effective post-pandemic teaching model, with the aim of enhancing the comprehensive abilities of medical students. Results showed that this case-based, integrated blended teaching model led to an increase in final exam scores, particularly in the proportion of high-scoring students across both cohorts. A significant score improvement was also observed in integrated module assessments. Survey results revealed that most students agreed the new model enhanced their self-directed learning, motivation, and classroom engagement, supporting personalized learning and knowledge integration. Consequently, this model may serve as a valuable template for post-pandemic course design in local medical universities.

## Introduction

The cultivation of outstanding medical professionals is evidently crucial, particularly with the rapid evolution of modern medical education. Undoubtedly, the COVID-19 pandemic has accelerated the need for educational institutions to integrate online learning into face-to-face tutorials, teaching, and training (1). Online learning offers several advantages, such as flexibility, cost-effectiveness, accessibility, learner satisfaction, and durable access to resources (2). However, it also presents disadvantages, including limited interactive feedback, social isolation, and technical disruptions caused by unexpected situations (2). Research indicates that a blended learning method (combining online and face-to-face instruction) is the most preferred option among both learners and institutions, followed by purely face-to-face and then purely online formats (3).

In 2008, Dave Cormier introduced the concept of Massive Open Online Courses (MOOCs) (4). Alongside international platforms like Coursera and edX, China has established its own MOOC ecosystems, including XuetangX and the Chinese University MOOC Alliance, to serve Chinese-speaking learners (5, 6). This development is particularly evident in higher education, where an increasing array of platforms dedicated to medical courses has emerged (7), playing a key role in sustaining educational activities during and after the pandemic.

The ultimate goal of medical education is to cultivate competent physicians with a solid knowledge base, positive learning attitudes, well-developed clinical reasoning, the ability to integrate knowledge, and high levels of engagement (8). Meanwhile, the knowledge of basic sciences and its relevance or utility in clinical situations should also be emphasized. Case-based learning (CBL) addresses these needs by utilizing authentic clinical cases to foster greater student-teacher interaction and enhance the learning experience (9). As an andragogical paradigm, CBL integrates multiple disciplines, encourages collaborative teamwork, and cultivates analytical and problem-solving skills, thereby promoting constructive knowledge assimilation (9, 10). These attributes underscore its significant role in medical education.

In terms of integration cultivation, researchers has reported several ways of improving the integration of knowledge effectively in basic medicine education process with the sustainable development of medical and health care (11). Several medical schools have employed the model of organ system integration teaching reform of education and teaching methods which centered on students and focused on self-directed learning by way of vertical and horizontal curriculum integration (12). Additionally, the curriculum integration is presented in other forms of clinical disease-based courses from mechanism, clinical manifestations to clinical therapy strategies, also integrated cross-chapter courses based on different pathological process, enhancing the students’ ability to connect knowledge points (13, 14). Still, it remains unsure the most appropriate mode of integration.

In this study, a blended teaching format, which combined resources from the Chinese University MOOC platform with face-to-face instruction, was employed in the Pathophysiology course for third-year medical students at Guilin Medical University. The CBL and cross-chapter integration approaches implemented for the 2020 and 2021 cohorts were compared with the conventional blended teaching method used for the 2019 cohort. This comparison aimed to provide an effective approach and a novel perspective for teaching Pathophysiology that is suitable for local medical universities.

## Materials and methods

### Students

In Guilin Medical University, students take the Pathophysiology course in their third year (the fifth semester). The participants were undergraduate clinical students from three consecutive entry cohorts (grades of 2019, 2020, and 2021, representing the respective years of enrollment). The 596 students from the grade of 2019, who attended blended courses, were selected as the control group. In comparison, the 650 students from the grade of 2020 and the 714 students from the grade of 2021 participated in an enhanced course that combined blended methods with CBL, following the organ-system integration principle.

### Teaching plan

In recent years, the number of online courses in Chinese universities has grown significantly. Online teaching became especially widespread during the COVID-19 pandemic. Students across all three cohorts experienced the impact of the pandemic on their learning methods at different stages of their studies.

The Pathophysiology curriculum was consistent across the three grade levels, with a total of 39 theoretical credit hours. Each credit hour lasted 40 min, and each thematic unit comprised 3 credit hours (totaling 120 min). A large-class session typically had an enrollment of approximately 100 students. The instructional team for these sessions consisted of one or two primary consistent lecturers, assisted by three teaching assistants from the discipline.

For the grade of 2019, a blended teaching model was implemented using the Chinese University MOOC platform. This model comprised three phases: (1) pre-class self-study, involving watching assigned MOOC videos, reading textbook chapters, and reviewing instructor-prepared slides; (2) an in-class quiz (15 min, 20 points) administered via the MOOC platform at the beginning of each session, followed by instructor feedback on common errors; and (3) a subsequent lecture covering the remaining core content.

For the grades of 2020 and 2021, the Pathophysiology curriculum was restructured into four organ-system integrated modules: Non-Integrated Fundamentals (introduction to disease, fever, stress, ischemia–reperfusion injury, coagulation and anticoagulation disturbances, hepatic insufficiency), the Cardiovascular System (shock, cardiac insufficiency), the Respiratory System (hypoxia, respiratory insufficiency), and the Urinary System (water and electrolyte balance and imbalance, acid–base balance and imbalance, renal insufficiency). Their teaching model retained the pre-class online self-study and quiz components but replaced the post-quiz lecture with a CBL session. During these face-to-face CBL sessions, students, working in groups, analyzed provided clinical cases (print or video) to solve instructor-designed questions that linked theory to practice. Group conclusions were presented by volunteers and discussed with peers and instructors. Each session concluded with an instructor-led synthesis of key points, performance assessment, and clarification of difficult topics (20 min).

Course assessment comprised in-class quizzes and periodic exams. These included multiple-choice, short-answer, and case analysis questions, and were administered both online and offline. The final exam was an offline, closed-book test designed by the university’s Academic Affairs Office using the Test Database of Chinese Medical Education and subsequently reviewed by experienced faculty members in Pathophysiology.

### Questionnaire

To evaluate students’ perceptions of the teaching model that combined blended learning and CBL based on cross-chapter integration, an anonymous questionnaire was administered based on previously established instruments. The questionnaire comprised seven items rated on a 5-point Likert scale (strongly disagree, disagree, neutral, agree, strongly agree) and three multiple-choice items.

### Statistics

Data were analyzed with Microsoft Excel and SPSS 20.0. Final exam scores are expressed as mean ± SD and were compared using one-way ANOVA with LSD post-hoc tests. Mastery rates and survey results are presented as percentages and were compared using the Chi-square test. A *p* < 0.05 was considered statistically significant.

## Results

### Comparison of student performance under different teaching methods

**Figure 1:**
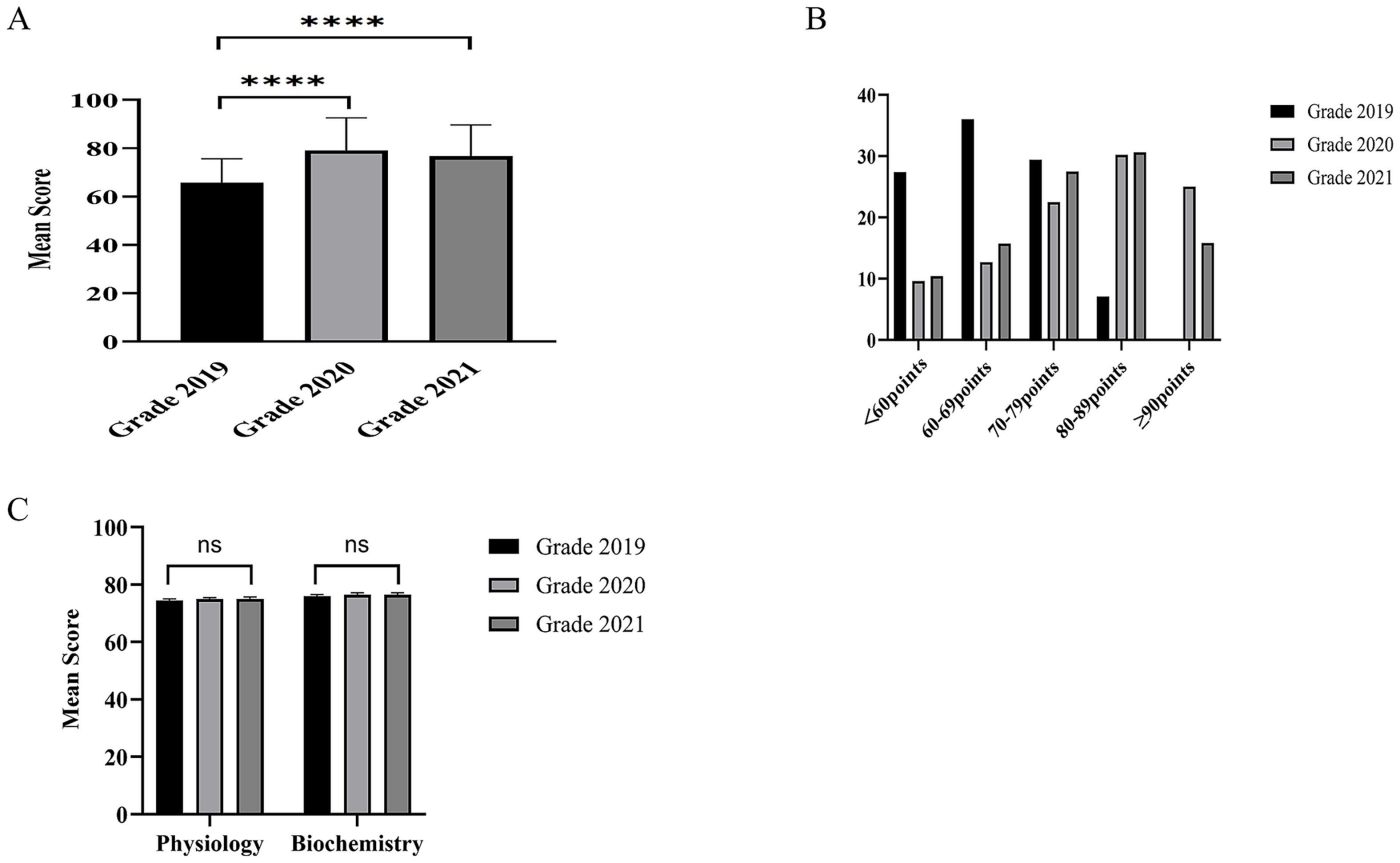
Comparison of student performance between different teaching methods. **(A)** Average scores of the pathophysiology final examination across the grades of 2019, 2020, and 2021. **(B)** Distribution of the final exam scores across the grades of 2019, 2020, and 2021. **(C)** Average scores of the physiology and biochemistry final examination across the grades of 2019, 2020, and 2021. ^****^p < 0.0001 vs. the 2019 grade, ns, not significant.

The grade of 2019 was taught using blended methods, while the 2020 and 2021 grades received a modified curriculum that integrated CBL within an organ-system integration principle alongside the established blended approach. Assessment was conducted using a standardized final exam, comprising multiple-choice questions from the Test Database of Chinese Medical Education, developed by the People’s Medical Publishing House. Results revealed that the latter two cohorts achieved significantly higher mean scores compared to the 2019 cohort (Figure 1A). Score distribution analysis further showed a marked shift toward higher performance in the intervention groups (Figure 1B). Specifically, the proportion of students scoring below 70 points was substantially lower in the 2020 and 2021 cohorts. In contrast, the proportion achieving scores of 80 or above was notably higher (Figure 1B), indicating that the integrated CBL curriculum was associated with a greater concentration of students in the upper performance tiers. Further analysis of the admission exam scores and the grades of preceding courses (Physiology and Biochemistry) closely related to Pathophysiology across these three grades showed no significant differences among them (Supplementary Table 1; Figure 1C). Therefore, the changes in scores and their distribution highlight the intervention’s impact on improving overall cohort performance.

### Assessment of blended methods and CBL based on the organ-system integration principle on curriculum learning

The reformed teaching plan integrated the chapters in organ-system principle combined with CBL in 2020 and 2021 grades. Accordingly, we observed students’ performance in different modules. In the non-integrated module, the correct answer rates of the three grades were 67.88, 80.98, 75.5%, respectively. In the cardiovascular module, the rates were 65.3, 78.27, 80.26%, respectively. In the pulmonary module, the rates were 59.62, 66.73, 74.00% separately, and in the urinary module, the rates were 66.75, 73.23, 78.74% individually. Summarily, compared with the 2019 grade, students from both 2020 and 2021 grades acquired significant higher scores in all the involved modules (Table 1).

**Table 1:** The mastery rate comparison among the 2019, 2020, 2021 grades in each module.

Compared years^a,b^	Non-integrated	Cardiovascular	Pulmonary	Urinary
2019 vs. 2020	27.69^***^	26.26^***^	6.95^**^	6.18^*^
2019 vs. 2021	9.17^**^	37.38^***^	30.60^***^	23.63^***^
2020 vs. 2021	5.87^*^	0.78	8.44^**^	5.62^**^

^a^Values in the table are chi-square values (χ^2^). ^b^Significance is denoted as follows: *p < 0.05, **p < 0.01, ***p < 0.001.

In this study, a separation of teaching and assessment was implemented. Consistency was maintained across syllabus coverage (understand, 13%; familiarize, 30%; master, 55%; superclass, 2%), question type composition (A1, 100 single-sentence best-choice questions, focus on foundational knowledge and recall; A2, 35 case summary best-choice questions, assessing clinical reasoning through case-integrated judgment; B, 15 standard matching questions, evaluating ability to differentiate between similar concepts). Meanwhile, the overall difficulty of the assessment papers was reassessed, revealing no change (Supplementary Table 2). Furthermore, the reliability coefficients of the exam papers were 0.89, 0.96, and 0.95, respectively, all exceeding 0.8. However, compared with the 2019 grade, the correct answer rates for most items were significantly higher in the 2020 and 2021 grades (Tables 2, 3). Notable improvements were observed in the interpretation mastery rate (62.96, 76.89, 79.82%) (Supplementary Table 3) and in type A2 questions, which focus on the clinical application of knowledge (65.71, 72.25, 79.82%) (Supplementary Table 4). These findings suggest enhanced problem-solving and application abilities among students in the 2020 and 2021 cohorts.

**Table 2:** The mastery rate comparison among the 2019, 2020, 2021 grades in cognitive levels.

Compared years^a,b^	Knowledge recall	Interpretation	Problem-solving
2019 vs. 2020	13.97^**^	24.92^***^	5.86^*^
2019 vs. 2021	9.97^**^	40.67^***^	9.35^**^
2020 vs. 2021	0.47	1.70	0.35

^a^Values in the table are chi-square values (χ^2^). ^b^Significance is denoted as follows: *p < 0.05, **p < 0.01, ***p < 0.001.

**Table 3:** The mastery rate comparison among the 2019, 2020, 2021 grades in question types.

Compared Years^a,b^	A1	A2	B
2019 vs. 2020	12.63^***^	6.23^*^	46.42^***^
2019 vs. 2021	6.65^**^	28.13^***^	113.21^***^
2020 vs. 2021	1.18	7.93^**^	14.77^***^

^a^Values in the table are chi-square values (χ^2^). ^b^Significance is denoted as follows: *p < 0.05, **p < 0.01, ***p < 0.001.

### Evaluation and suggestions for this teaching plan from students’ perspective

**Figure 2:**
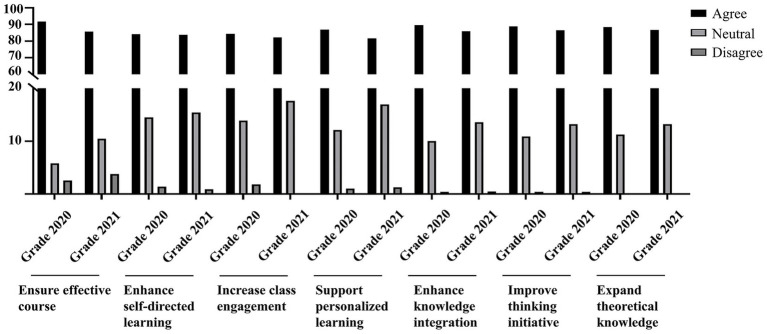
Responses of the students to the transformed teaching plan. The Cronbach’s alpha values were 0.954, 0.945, and 0.953, respectively, and the KMO values were 0.951, 0.931, and 0.958.

Students showed positive attitudes toward the integrated teaching model, which combined blended learning with CBL based on the organ-system integration principle (Figure 2; Supplementary Table 5). A majority of students indicated that the Pathophysiology course was well organized (91.55% of the 2020 cohort and 85.71% of the 2021 cohort). Furthermore, 84.10% of the 2020 grade and 83.71% of the 2021 grade reported that the teaching model enhanced their self-directed learning abilities. Similarly, 84.31 and 82.15% of students from the 2020 and 2021 grades, respectively, agreed that the approach increased their motivation and willingness to participate in class, and believed it better supported personalized learning (86.92 and 81.70%, respectively). A high proportion of students also acknowledged the model’s role in promoting the integration of basic knowledge with clinical practice (89.54 and 85.93%, respectively) and strengthening theoretical connections. Moreover, 88.73% of the 2020 cohort and 86.39% of the 2021 cohort felt that the approach improved their subjective initiative and comprehensive integrative thinking skills. Finally, 88.53% of students in the 2020 grade and 86.61% in the 2021 grade agreed that the reformed curriculum expanded the breadth and depth of their theoretical knowledge.

### Observation of students’ self-study before class

**Figure 3:**
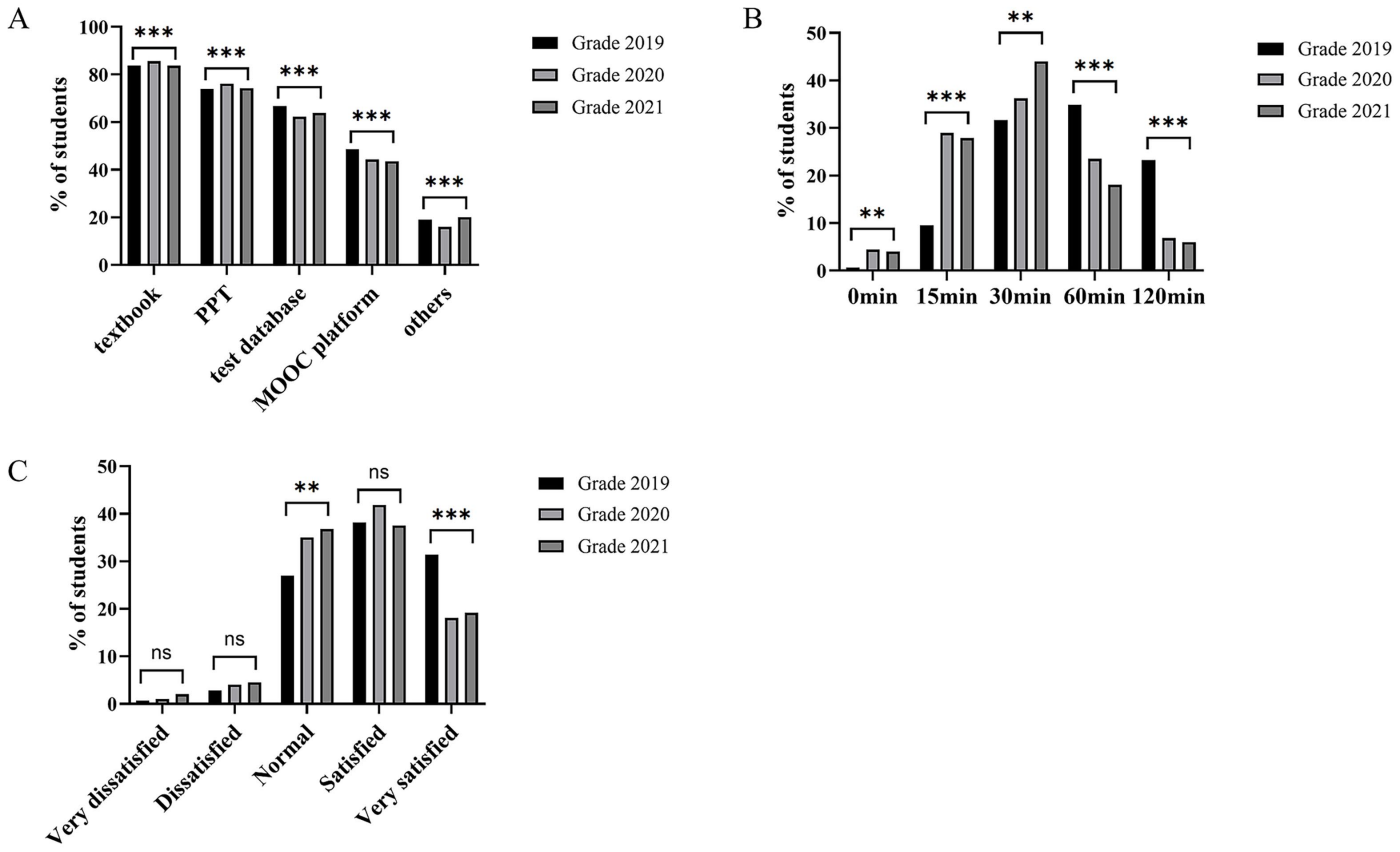
Observation of students’ self-directed learning engagement before class. **(A)** Preferred preview materials rated by students. **(B)** Distribution of time spent on pre-class self-study. **(C)** Students’ self-perceived achievement of pre-class learning goals. ^**^p < 0.01, ***p < 0.001, ns, not significant.

Some pre-class learning activities were implemented under the reformed teaching model, including previewing relevant content on the MOOC platform, reviewing textbooks, and studying PPT slides (Figure 3; Supplementary Table 5). As a result, students gained a better understanding of the course materials and engaged more effectively in case discussions. Upon completing these tasks, students were surveyed via Wenjuanxing, an online survey platform. Regarding preview materials, the majority of students identified the textbook as their primary resource (83.72% in 2019, 85.60% in 2020, and 83.71% in 2021), followed by PPT slides, the test database of Chinese Medical Education, and the MOOC platform (Figure 3A). In terms of time spent on pre-class learning, 34.88% of students in the 2019 cohort spent 60 min completing preview tasks. In contrast, lower proportions of students in the 2020 and 2021 cohorts (23.54 and 18.08%, respectively) spent between 30 and 60 min (Figure 3B), likely reflecting increased efficiency and adaptation influenced by the pandemic context. When asked about achieving their self-learning goals, 69.54% of students in the 2019 cohort believed they had accomplished their objectives (Figure 3C). However, the proportion of students in the 2020 and 2021 cohorts declined to 59.96 and 56.70%, respectively.

## Discussion

In this study, the 2019 cohort received instruction through pre-class self-study, pre-class quizzes, and traditional lectures. In contrast, the 2020 and 2021 cohorts experienced a reorganized curriculum that incorporated pre-class self-study, pre-class quizzes, and CBL. As has been described, no significant differences in admission or prerequisite scores were found among the three groups, with key variables such as student intake, instructors, and exam design held constant. The results demonstrated that the final examination scores of the experimental cohorts (2020 and 2021) were higher than those of the control cohort (2019), with a considerably greater proportion of students achieving scores≥80. These findings indicate the enhanced effectiveness of a teaching model that combines blended learning with CBL, grounded in the principle of organ-system integration. This trend aligns with, but is more pronounced than, the outcomes of our earlier study conducted with a subset of students prior to the pandemic (15).

Traditional lectures were reported to promote passive learning, limited clinical reasoning development, and low engagement (16, 17). The widespread adoption of online learning during the pandemic offered an alternative (1, 7). However, successful online education depends heavily on self-motivated learners and high-quality resources and exhibits other shortcomings (2, 17). In response, a blended approach that combines face-to-face and online elements has been widely advocated as a more balanced and effective model for medical education (18, 19). Notably, the 2019 cohort exhibited a lower proportion of students scoring ≥80, potentially due to pandemic-driven reductions in essential face-to-face interaction.

Here, data showed that the 2020 and 2021 cohorts spent less time on pre-class activities compared with the 2019 cohort. This reduction may be attributed to enhanced student self-directed learning ability, improved availability and quality of curricular resources, access to external platforms. It is also noteworthy that despite the availability of diverse online resources, students consistently rated textbooks as their preferred non-interactive learning material (documents, pre-recorded presentations, and audiovisual files), suggesting a need for further exploration into the design of pre-class learning materials.

Pathophysiology serves as a critical bridge between basic and clinical medicine. Traditional teaching, however, often segments content into isolated pathological processes and organ dysfunctions, making it challenging for students to integrate this knowledge with related clinical courses (11). Curriculum integration (horizontal, vertical, spiral) has been recognized to enhance long-term knowledge retention and clinical relevance (20, 21). Nevertheless, its implementation faces obstacles such as limited resources and institutional constraints, especially in local medical schools with large enrollment size.

In the present study, we redesigned Pathophysiology course to integrated modules (cardiovascular, respiratory, renal) alongside a non-integrated module. Compared to the 2019 cohort, student performance significantly improved in the integrated modules for the 2020 and 2021 cohorts, indicating that cross-chapter integration strengthens learning outcomes. Student feedback further confirmed that this approach improved the connection between basic mechanisms and clinical practice, fostering integrated clinical thinking. These findings suggest that cross-chapter structured content integration, rather than the extent of integration alone, can effectively develop the synthesizing skills essential for clinical competency, identical to previous study (9, 11).

Medical education is shifting from traditional lectures toward active, student-centered approaches like PBL and CBL, which aim to improve learning effectiveness (22–24). CBL, in particular, employs realistic cases to enhance clinical competence, conceptual integration, and relevance (25, 26). Studies suggest that combining CBL with lectures in small groups is more effective than individual CBL activities (9, 11). In this study, analysis of final exam questions, including difficulty, question type and cognitive level, showed no significant difference in overall exam difficulty across cohorts. However, mastery rate of type A2 questions (which assess clinical application and problem-solving) was higher in the 2020 and 2021 cohorts, consistent with previous findings (15). Student feedback indicated that the CBL-based reform enhanced self-directed learning, engagement, motivation, and depth of theoretical knowledge. Most of the students felt the course was well organized and met learning objectives. However, some students, however, were dissatisfied with their self-learning outcomes and indicated a need for more learning resources, support, and strategic guidance. This dissatisfaction likely stems from a growing awareness of the limitations of self-directed learning and heightened expectations for learning outcomes.

Interestingly, the integration of artificial intelligence (AI) into medical education has accelerated in recent years, expanding beyond clinical practice into teaching and learning, with AI competencies now recognized as essential skills for medical students (27, 28). In our study, the 2020 and 2021 cohorts spent less time on pre-class tasks than the 2019 cohort. Notably, during CBL sessions, responses from the 2021 grade demonstrated marked improvements in length, rigor, and accuracy. It is hypothesized that these enhancements may be associated with the use of AI tools such as Doubao and ChatGPT, although this observation necessitates validation through measured data. Collectively, these findings suggest that AI tools may have significant potential in Pathophysiology education, though additional efforts are required to optimize their use and ensure accuracy.

In this study, we developed a blended Pathophysiology model that integrates CBL with organ-system teaching. This model reduced content overlap and improved connections between theory and practice. Student feedback and assessment results showed it enhanced engagement, motivation, and integrated learning.

In summary, the adoption of CBL coupled with cross-chapter integration is recommended for Pathophysiology curriculum. This approach effectively supports knowledge integration, personalized learning, and long-term competency development, which is especially relevant to post-pandemic education. It is important to note that the findings are based on a single-institution study. Therefore, longer-term educational outcomes warrant further investigation. Additionally, factors such as annual fluctuations in student preparedness will be considered in future research.

## Data Availability

The original contributions presented in the study are included in the article/Supplementary material, further inquiries can be directed to the corresponding author.
