# Supplementary material for: The post-pandemic transformation in Pathophysiology teaching strategies
Source: Front Med (Lausanne). 2026 Apr 22;13:1738205. doi: 10.3389/fmed.2026.1738205 (PMC13143679; doi:10.3389/fmed.2026.1738205)
Supplement: Supplementary file 1 [file Table_1.docx]

**Suppl. Table 1 One-way ANOVA of Z-scored admission exam scores across three grades (N = 1909)**

| Source | SS | df | MS | F | p |
| --- | --- | --- | --- | --- | --- |
| Between Groups | 0.000 | 2 | 0.000 | 0.000 | 1.000 |
| Within Groups | 1841.000 | 1906 | 0.966 |  |  |
| Total | 1841.000 | 1908 |  |  |  |

Note: SS = sum of squares; MS = mean square. All assessment scores were converted to z-scores prior to analysis. The results indicate no significant difference among the three grades.
