# Supplementary material for: The post-pandemic transformation in Pathophysiology teaching strategies
Source: Front Med (Lausanne). 2026 Apr 22;13:1738205. doi: 10.3389/fmed.2026.1738205 (PMC13143679; doi:10.3389/fmed.2026.1738205)
Supplement: Supplementary file 2 [file Table_2.docx]

**Suppl. Table 2 The difficult level comparison of the final paper among the 2019, 2020, 2021 grades**

| Year (N) | No  difficulty | Mild  difficulty | Moderate  difficulty | Severe  difficulty | Profound  difficulty |
| --- | --- | --- | --- | --- | --- |
| 2019 (n = 150) | 21 (14.0%) | 20 (13.3%) | 84 (56.0%) | 16 (10.7%) | 9 (6.0%) |
| 2020 (n = 150) | 27 (18.0%) | 25(16.7%) | 80 (53.3%) | 12 (8.0%) | 6 (4.0%) |
| 2021 (n = 150) | 19 (12.7%) | 18 (12.0%) | 79 (52.7%) | 23 (15.3%) | 11 (7.3%) |

Note: Data are presented as number of students (percentage). Chi-square test: χ²(8) = 8.072, p = 0.426. Items were classified into five using the following threshold: >0.9 (No difficulty), 0.7-0.9 (mild difficulty), 0.5-0.69 (moderate difficulty), 0.3-0.49 (severe difficulty), and <0.3 (profound difficulty).
