# Supplementary material for: The post-pandemic transformation in Pathophysiology teaching strategies
Source: Front Med (Lausanne). 2026 Apr 22;13:1738205. doi: 10.3389/fmed.2026.1738205 (PMC13143679; doi:10.3389/fmed.2026.1738205)
Supplement: Supplementary file 3 [file Table_3.docx]

**Suppl. Table 3 The mastery rate comparison among the 2019, 2020, 2021 classes in cognitive levels**

| Congenital level | 2019 | 2020 | 2021 |
| --- | --- | --- | --- |
| Knowledge recall | 403 (67.65) | 501 (77.01) | 539 (75.47) |
| Interpretation | 375 (62.96) | 500 (76.89) | 570 (79.82) |
| Problem-solving | 400 (67.11) | 477 (73.32) | 534 (74.76) |
| Total Responses (n) | 596 | 650 | 714 |

Note: The table presents the number of correct responses and the corresponding percentage; n indicates the total number of students.
