# Supplementary material for: The post-pandemic transformation in Pathophysiology teaching strategies
Source: Front Med (Lausanne). 2026 Apr 22;13:1738205. doi: 10.3389/fmed.2026.1738205 (PMC13143679; doi:10.3389/fmed.2026.1738205)
Supplement: Supplementary file 4 [file Table_4.docx]

**Suppl. Table 4 The mastery rate comparison among the 2019, 2020, 2021 grades in question types**

| Question type | 2019 | 2020 | 2021 |
| --- | --- | --- | --- |
| A1 | 411 (69.00) | 506 (77.84) | 538 (75.47) |
| A2 | 392 (65.71) | 470 (72.25) | 563 (79.82) |
| B | 319 (53.55) | 469 (72.21) | 578 (74.76) |
| Total Responses (n) | 596 | 650 | 714 |

Note: The table presents the number of correct responses and the corresponding percentage; n indicates the total number of students.
