# Supplementary material for: The post-pandemic transformation in Pathophysiology teaching strategies
Source: Front Med (Lausanne). 2026 Apr 22;13:1738205. doi: 10.3389/fmed.2026.1738205 (PMC13143679; doi:10.3389/fmed.2026.1738205)
Supplement: Supplementary file 5 [file Table_5.docx]

**Questionnaire Survey on Online and Offline Teaching of Pathophysiology-Clinical Medicine Undergraduate (Grade 2019)**

**Question 1: Complete the self-study task. What are your favorite learning resources? [Multiple Choice Question]**

| Option | subtotal | proportion |
| --- | --- | --- |
| textbook | 360 | 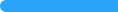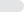83.72% |
| PPT | 318 | 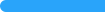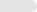73.95% |
| test database | 287 | 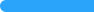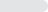66.74% |
| MOOC platform | 209 | 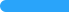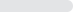48.6% |
| others | 82 | 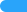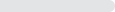19.07% |
| Number of valid responses for this question | 430 |  |

**Question 2: The actual time you spent on the pre-class self-study task is [Single Choice Question]**

| Option | subtotal | proportion |
| --- | --- | --- |
| 0min | 3 | 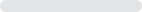0.7% |
| 15min | 41 | 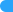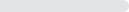9.53% |
| 30min | 136 | 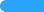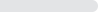31.63% |
| 60min | 150 | 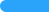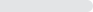34.88% |
| 120min | 100 | 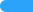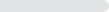23.26% |
| Number of valid responses for this question | 430 |  |

**Question 3: Pre-class tasks typically cover easily understandable content within the chapter, such as an overview of fever, etiology, and classification of shock. Do you believe self-study can achieve the learning objectives (are you satisfied with your self-study outcomes)? [Single Choice Question]**

| Option | subtotal | proportion |
| --- | --- | --- |
| Very dissatisfied | 3 | 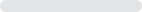0.7% |
| Dissatisfied | 12 | 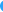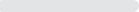2.79% |
| Normal | 116 | 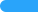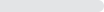26.98% |
| Satisfied | 164 | 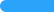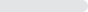38.14% |
| Very satisfied | 135 | 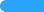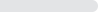31.4% |
| Number of valid responses for this question | 430 |  |

**Questionnaire Survey on Online and Offline Teaching of Pathophysiology-Clinical Medicine Undergraduate Classes 1, 2, 7, 8, 9, and 10 (Grade 2020)**

**Question 1: Complete the self-study task. What are your favorite learning resources? [Ranking question]**

| Option | Top Priority | Second Priority | Third Priority | Fourth Priority | Fifth Priority | Subtotal |
| --- | --- | --- | --- | --- | --- | --- |
| textbook | 161(55.14%) | 68(23.29%) | 40(13.7%) | 21(7.19%) | 2(0.68%) | 292 |
| PPT | 54(18.62%) | 143(49.31%) | 63(21.72%) | 28(9.66%) | 2(0.69%) | 290 |
| test database | 38(13.24%) | 44(15.33%) | 142(49.48%) | 59(20.56%) | 4(1.39%) | 287 |
| MOOC platform | 30(11.67%) | 26(10.12%) | 34(13.23%) | 153(59.53%) | 14(5.45%) | 257 |
| others | 9(8.33%) | 11(10.19%) | 13(12.04%) | 31(28.7%) | 44(40.74%) | 108 |

**Question 2: The actual time you spent on the pre-class self-study task is [Single Choice Question]**

| Option | subtotal | proportion |
| --- | --- | --- |
| 0min | 16 | 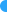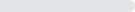5.48% |
| 15min | 89 | 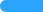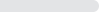30.48% |
| 30min | 109 | 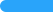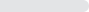37.33% |
| 60min | 58 | 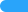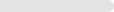19.86% |
| 120min | 20 | 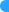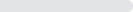6.85% |
| Number of valid responses for this question | 292 |  |

**Question 3: Pre-class tasks typically cover easily understandable content within the chapter, such as an overview of fever, etiology, and classification of shock. Do you believe self-study can achieve the learning objectives (are you satisfied with your self-study outcomes)? [Single Choice Question]**

| Option | subtotal | proportion |
| --- | --- | --- |
| Very dissatisfied | 3 | 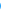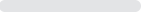1.03% |
| Dissatisfied | 11 | 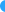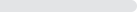3.77% |
| Normal | 102 | 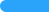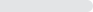34.93% |
| Satisfied | 129 | 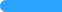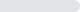44.18% |
| Very satisfied | 47 | 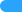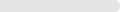16.1% |
| Number of valid responses for this question | 292 |  |

**Question 4: Be satisfied with the teaching plan of Pathophysiology [Single Choice Question]**

| Option | subtotal | proportion |
| --- | --- | --- |
| Strongly disagree | 6 | 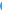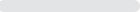2.05% |
| Disagree | 2 | 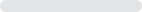0.68% |
| Neutral | 20 | 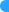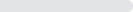6.85% |
| Agree | 148 | 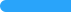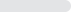50.68% |
| Strongly agree | 116 | 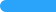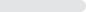39.73% |
| Number of valid responses for this question | 292 |  |

**Question 5: Enhance your self-directed learning [Single Choice Question]**

| Option | subtotal | proportion |
| --- | --- | --- |
| Strongly disagree | 3 | 1.03% |
| Disagree | 3 | 1.03% |
| Neutral | 41 | 14.04% |
| Agree | 158 | 54.11% |
| Strongly agree | 87 | 29.79% |

**Question 6: Increase your motivation and willingness to engage in the class learning [Single Choice Question]**

| Option | subtotal | proportion |
| --- | --- | --- |
| Strongly disagree | 4 | 1.37% |
| Disagree | 3 | 1.03% |
| Neutral | 40 | 13.7% |
| Agree | 150 | 51.37% |
| Strongly agree | 95 | 32.53% |

**Question 7: Support your personalized learning better [Single Choice Question]**

| Option | subtotal | proportion |
| --- | --- | --- |
| Strongly disagree | 2 | 0.68% |
| Disagree | 0 | 0% |
| Neutral | 34 | 11.64% |
| Agree | 157 | 53.77% |
| Strongly agree | 99 | 33.9% |

**Question 8: Enhance the integration of basic knowledge with clinical practice and theoretical connections [Single Choice Question]**

| Option | subtotal | proportion |
| --- | --- | --- |
| Strongly disagree | 1 | 0.34% |
| Disagree | 0 | 0% |
| Neutral | 28 | 9.59% |
| Agree | 161 | 55.14% |
| Strongly agree | 102 | 34.93% |

**Question 9: Improve your subjective initiative and comprehensive thinking ability [Single Choice Question]**

| Option | subtotal | proportion |
| --- | --- | --- |
| Strongly disagree | 1 | 0.34% |
| Disagree | 0 | 0% |
| Neutral | 29 | 9.93% |
| Agree | 160 | 54.79% |
| Strongly agree | 102 | 34.93% |

**Question 10: Expand the breadth and depth of your theoretical knowledge [Single Choice Question]**

| Option | subtotal | proportion |
| --- | --- | --- |
| Strongly disagree | 1 | 0.34% |
| Disagree | 0 | 0% |
| Neutral | 30 | 10.27% |
| Agree | 162 | 55.48% |
| Strongly agree | 99 | 33.9% |

**Questionnaire Survey on Online and Offline Teaching in Pathophysiology-Clinical Medicine Undergraduate Classes 3, 4, 5, and 6 (Grade 2020)**

**Question 1: Complete the self-study task. What are your favorite learning resources? [Ranking question]**

| Option | Top Priority | Second Priority | Third Priority | Fourth Priority | Fifth Priority | Subtotal |
| --- | --- | --- | --- | --- | --- | --- |
| textbook | 118(57.56%) | 50(24.39%) | 20(9.76%) | 16(7.8%) | 1(0.49%) | 205 |
| PPT | 48(23.65%) | 105(51.72%) | 37(18.23%) | 12(5.91%) | 1(0.49%) | 203 |
| test database | 22(10.95%) | 31(15.42%) | 107(53.23%) | 39(19.4%) | 2(1%) | 201 |
| MOOC platform | 13(7.43%) | 13(7.43%) | 26(14.86%) | 114(65.14%) | 9(5.14%) | 175 |
| others | 4(5.26%) | 6(7.89%) | 15(19.74%) | 24(31.58%) | 27(35.53%) | 76 |

**Question 2: The actual time you spent on the pre-class self-study task is [Single Choice Question]**

| Option | Subtotal | proportion |
| --- | --- | --- |
| 0min | 6 | 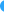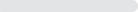2.93% |
| 15min | 55 | 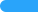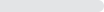26.83% |
| 30min | 71 | 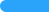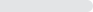34.63% |
| 60min | 59 | 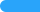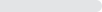28.78% |
| 120min | 14 | 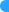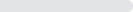6.83% |
| Number of valid responses for this question | 205 |  |

**Question 3: Pre-class tasks typically cover easily understandable content within the chapter, such as an overview of fever, etiology, and classification of shock. Do you believe self-study can achieve the learning objectives (are you satisfied with your self-study outcomes)? [Single Choice Question]**

| Option | subtotal | proportion |
| --- | --- | --- |
| Very dissatisfied | 2 | 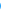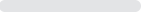0.98% |
| Dissatisfied | 9 | 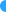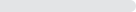4.39% |
| Normal | 72 | 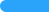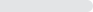35.12% |
| Satisfied | 79 | 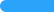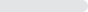38.54% |
| Very satisfied | 43 | 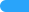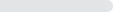20.98% |
| Number of valid responses for this question | 205 |  |

**Question 4: Be satisfied with the teaching plan of Pathophysiology [Single Choice Question]**

| Option | subtotal | proportion |
| --- | --- | --- |
| Strongly disagree | 5 | 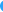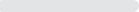2.44% |
| Disagree | 0 | 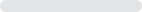0% |
| Neutral | 9 | 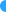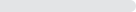4.39% |
| Agree | 96 | 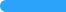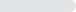46.83% |
| Strongly agree | 95 | 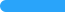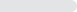46.34% |
| Number of valid responses for this question | 205 |  |

**Question 5: Enhance your self-directed learning [Single Choice Question]**

| Option | subtotal | proportion |
| --- | --- | --- |
| Strongly disagree | 0 | 0% |
| Disagree | 1 | 0.49% |
| Neutral | 31 | 15.12% |
| Agree | 104 | 50.73% |
| Strongly agree | 69 | 33.66% |

**Question 6: Increase your motivation and willingness to engage in the class learning [Single Choice Question]**

| Option | subtotal | proportion |
| --- | --- | --- |
| Strongly disagree | 1 | 0.49% |
| Disagree | 1 | 0.49% |
| Neutral | 29 | 14.15% |
| Agree | 108 | 52.68% |
| Strongly agree | 66 | 32.2% |

**Question 7: Support personalized learning better [Single Choice Question]**

| Option | subtotal | proportion |
| --- | --- | --- |
| Strongly disagree | 2 | 0.98% |
| Disagree | 1 | 0.49% |
| Neutral | 26 | 12.68% |
| Agree | 104 | 50.73% |
| Strongly agree | 72 | 35.12% |

**Question 8: Enhance the integration of basic knowledge with clinical practice and theoretical connections [Single Choice Question]**

| Option | subtotal | proportion |
| --- | --- | --- |
| Strongly disagree | 0 | 0% |
| Disagree | 1 | 0.49% |
| Neutral | 22 | 10.73% |
| Agree | 111 | 54.15% |
| Strongly agree | 71 | 34.63% |

**Question 9: Improve your subjective initiative and comprehensive thinking ability [Single Choice Question]**

| Option | subtotal | proportion |
| --- | --- | --- |
| Strongly disagree | 1 | 0.49% |
| Disagree | 0 | 0% |
| Neutral | 25 | 12.2% |
| Agree | 113 | 55.12% |
| Strongly agree | 66 | 32.2% |

**Question 10: Expand the breadth and depth of your theoretical knowledge [Single Choice Question]**

| Option | subtotal | proportion |
| --- | --- | --- |
| Strongly disagree | 0 | 0% |
| Disagree | 0 | 0% |
| Neutral | 26 | 12.68% |
| Agree | 113 | 55.12% |
| Strongly agree | 66 | 32.2% |

**Questionnaire Survey on Online and Offline Teaching of Pathophysiology-Clinical Medicine Undergraduate (Grade 2021)**

**Question 1: Complete the self-study task. What are your favorite learning resources? [Ranking question]**

| Option |  | Top Priority | Second Priority | Third Priority | Fourth Priority | Fifth Priority | Subtotal |
| --- | --- | --- | --- | --- | --- | --- | --- |
| textbook |  | 221(49.44%) | 126(28.19%) | 66(14.77%) | 32(7.16%) | 2(0.45%) | 447 |
| PPT |  | 95(21.35%) | 195(43.82%) | 104(23.37%) | 48(10.79%) | 3(0.67%) | 445 |
| test database |  | 71(16.4%) | 85(19.63%) | 191(44.11%) | 75(17.32%) | 11(2.54%) | 433 |
| MOOC platform |  | 32(8.02%) | 28(7.02%) | 64(16.04%) | 239(59.9%) | 36(9.02%) | 399 |
| others |  | 29(15.34%) | 14(7.41%) | 23(12.17%) | 54(28.57%) | 69(36.51%) | 189 |

**Question 2: The actual time you spent on the pre-class self-study task is [Single Choice Question]**

| Option | Subtotal | proportion |
| --- | --- | --- |
| 0min | 18 | 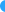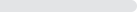4.02% |
| 15min | 125 | 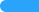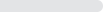27.9% |
| 30min | 197 | 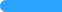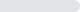43.97% |
| 60min | 81 | 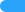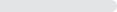18.08% |
| 120min | 27 | 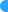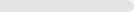6.03% |
| Number of valid responses for this question | 448 |  |

**Question 3: Pre-class tasks typically cover easily understandable content within the chapter, such as an overview of fever, etiology, and classification of shock. Do you believe self-study can achieve the learning objectives (are you satisfied with your self-study outcomes)? [Single Choice Question]**

| Option | Subtotal | proportion |
| --- | --- | --- |
| Very dissatisfied | 9 | 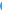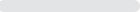2.01% |
| Dissatisfied | 20 | 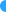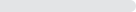4.46% |
| Normal | 165 | 36.83% |
| Satisfied | 168 | 37.5% |
| Very satisfied | 86 | 19.2% |
| Number of valid responses for this question | 448 |  |

**Question 4: Be satisfied with the teaching plan of Pathophysiology [Single Choice Question]**

| Option | subtotal | proportion |
| --- | --- | --- |
| Strongly disagree | 12 | 2.68% |
| Disagree | 5 | 1.12% |
| Neutral | 47 | 10.49% |
| Agree | 238 | 53.13% |
| Strongly agree | 146 | 32.59% |
| Number of valid responses for this question | 448 |  |

**Question 5: Enhance your self-directed learning [Single Choice Question]**

| Option | subtotal | proportion |
| --- | --- | --- |
| Strongly disagree | 1 | 0.22% |
| Disagree | 3 | 0.67% |
| Neutral | 69 | 15.4% |
| Agree | 256 | 57.14% |
| Strongly agree | 119 | 26.56% |

**Question 6: Increase your motivation and willingness to engage in the class learning [Single Choice Question]**

| Option | subtotal | proportion |
| --- | --- | --- |
| Strongly disagree | 1 | 0.22% |
| Disagree | 0 | 0% |
| Neutral | 79 | 17.63% |
| Agree | 240 | 53.57% |
| Strongly agree | 128 | 28.57% |

**Question 7: Support personalized learning better [Single Choice Question]**

| Option | subtotal | proportion |
| --- | --- | --- |
| Strongly disagree | 0 | 0% |
| Disagree | 6 | 1.34% |
| Neutral | 76 | 16.96% |
| Agree | 234 | 52.23% |
| Strongly agree | 132 | 29.46% |

**Question 8: Enhance the integration of basic knowledge with clinical practice and theoretical connections [Single Choice Question]**

| Option | subtotal | proportion |
| --- | --- | --- |
| Strongly disagree | 0 | 0% |
| Disagree | 2 | 0.45% |
| Neutral | 61 | 13.62% |
| Agree | 254 | 56.7% |
| Strongly agree | 131 | 29.24% |

**Question 9: Improve your subjective initiative and comprehensive thinking ability [Single Choice Question]**

| Option | subtotal | proportion |
| --- | --- | --- |
| Strongly disagree | 1 | 0.22% |
| Disagree | 1 | 0.22% |
| Neutral | 59 | 13.17% |
| Agree | 255 | 56.92% |
| Strongly agree | 132 | 29.46% |

**Question 10: Expand the breadth and depth of your theoretical knowledge [Single Choice Question]**

| Option | subtotal | proportion |
| --- | --- | --- |
| Strongly disagree | 0 | 0% |
| Disagree | 1 | 0.22% |
| Neutral | 59 | 13.17% |
| Agree | 258 | 57.59% |
| Strongly agree | 130 | 29.02% |
